# Ability vs Compensation

**Published:** 1871-03

**Authors:** H. L. Sage


### ABILITY *vs.* COMPENSATION.

BY H. L. SAGE.

The measure of ability which one may possess, depends largely upon the improvement made of that already conferred. In one sense, then, it is natural, and it *may* be in another acquired, for all the powers of mind and body gain strength by cultivation, and thus it follows that man fulfills the end for which he was created, only by a proper use of his talents and apportunities. Success worthy of the name, can only be achieved by untiring diligence and God's blessing. Men with gifts of which the world takes cognizance, though endowed by the creator, more largely it may be than their fellows, have not been wont to remain in idleness. No one, however humble, but has gifts which need only to be polished to make them attract, in a measure at least, by the light that is in them. Even as great natural abilities, without a proper use, may amount to nothing, so one not so largely blessed in this respect, may, by earnest and persevering effort, attain to no small degree of excellence in his chosen profession. Of the whole matter, this is the sum: natural ability, whether possessed in a greater or less degree, can only be made efficient for the production of the best results, by honest purposes, resolutely maintained and carried out; by an abandonment of ease for the laborious journey, until life's limit is reached, or obstacles are overcome.

Were we to judge of one's ability by the value he puts upon his time, his services or his wares, it would not prove a difficult task. But, viewing it in the light of justice and of reason, the amount or measure of power or ability, that is necessary to *be*, and actually *is* exercised in the accomplishment of a given object, should regulate the rate of compensation for the service rendered.

Doubtless, there are cases in every one's practice, and perhaps not unfrequently, in which humanity should prompt to bear lightly upon the exchequer of this one or that one, to whom service is rendered; such will readily occur to every mind. But viewing it not in the abstract, should not the ability to perform a professional service in the best manner, if that ability develops "the harmony of a thing perfect in all its parts," or in other words, secures the best results, command a compensation commensurate with the skill employed?

But this is not all. There are other considerations which should have a bearing in the matter of charges in dental practice, for it is the latter to which more particular reference is here made. Every one knows of the harassing, health-impairing tendency of a long practice of our own specialty, owing to the confinement necessary to its faithful prosecution, the restrained and unhealthful postures attendant upon long operations, the small amount of time which the dentist can command for requisite physical culture and to other familiar causes. Is it surprising that so many constitutions yield to this continuous strain, after comparatively few years of practice?

The foregoing statements being true, I repeat, that compensation should be graduated in proportion to the ability *put forth* or the skill *employed*, the degree of success in the attainment of results aimed at, or the perfection acquired, with regard to a proper estimate of the time devoted to the object in view; perhaps, with some regard to the expense incurred, in proportion to the relative value to the patient of the service rendered; with regard to the effect produced upon the general health by the profession practiced or the business followed; in proportion to the probable or average time allowed from the latter consideration to gain a competency.

Leaving out of view the false estimates which are liable to be made, and which may result from an undue self-valuation, or an over-rating of one's ability, or of errors in judging of results, etc., (though in general, a commendable purpose to excel accompanies these failings,) it is plain, that the above principles are not carried out as they should be; and this follows from several causes—of which, the great and over-shadowing one is, the facility with which the public, taken as a whole, are misled and in the end injured, by the syren word "cheap." "Is he cheap," is the first question asked about the dentist, and not what is his reputation for honesty and ability.

With a great portion of the community, and not unfrequently those of whom we should expect better things, this word seemingly has a talismanic influence. "A low price, appeals to a low class" says Victor Hugo, and this generally speaking, is true, when applied in the practice of our profession, for this principle can not be carried out in connection with faithful well-doing.

It may be said, that a groveling and inferior class of dentists, appeal to a low class of people, through the medium of cheap advertising, etc. It is a sad feature, that the dental profession abounds with such clogs as the former.

But of the dentist who so lightly values his services; it is in the majority of cases true, that he acts according to a kind of instinct, and therefore honestly, owing probably, to a well grounded conviction of the comparative worthlessness of his services.

It is not at all complimentary to his skill or ability.

But allowing that he is in some cases conscientious in the discharge of his duties to his patients, he will then *desire* and *endeavor* to give them the benefit of the best operation, or if relating to artificial substitutes, those which, by a proper amount of skill and pains-taking, will best fulfill the indications required. Of course, the degree of success will be in proportion to the amount of ability *possessed* and *exercised*, aside from the want of care on the part of the patient, or the untoward and unfavorable natural obstacles which sometimes have a tendency to prevent permanent benefit from being realized.

Surely, he can not do himself or patients justice by a degrading submission to the cry which obtains with many, for professional services, at low figures.

There are several classes in every community—the cultivated, enlightened, clear headed and common sense portion, who can discriminate between the spurious and the genuine, and those, who, lacking these qualities, float like the froth upon the surface of the water, to be swayed by the first breeze of charlatanry that blows.

Would we have the public mind educated to the right standard? How important, first of all, that every element of progress should be seized upon and appropriated to the one permanent object—that of developing within *ourselves*, those faculties which may, by proper culture, be made to subserve the great ends for which they were given, and that we may, by reaching to the highest possible attainments in our rising profession, be enabled to place a correct estimate upon our labors for the public good.

